# Babesiosis due to the canine *Babesia microti*-like small piroplasm in dogs - first report from Portugal and possible vertical transmission

**DOI:** 10.1186/1756-3305-4-50

**Published:** 2011-04-13

**Authors:** Paula Brilhante Simões, Luís Cardoso, Manuela Araújo, Yael Yisaschar-Mekuzas, Gad Baneth

**Affiliations:** 1 Inno - Serviços Especializados em Veterinária, Braga, Portugal; 2 Department of Veterinary Sciences, University of Trás-os-Montes e Alto Douro, Vila Real, Portugal; 3 Parasite Disease Group, Instituto de Biologia Molecular e Celular, Universidade do Porto, Portugal; 4 School of Veterinary Medicine, Hebrew University of Jerusalem, Rehovot, Israel

## Abstract

**Background:**

Canine babesiosis (or piroplasmosis) is endemic in northern Portugal, but molecularly confirmed cases of infection with small piroplasms have not been reported in the country. Three German shepherd dogs - a bitch and its 2-month old pup and an unrelated male - clinically suspected of piroplasmosis were assessed for babesial infection.

**Results:**

Parasitemia with small piroplasms was detected by microscopy in two dogs. All three dogs were positive by PCR and the *Babesia microti*-like small piroplasm (syn. *Theileria annae*) was identified by DNA sequencing. These are the first confirmed cases of babesiosis caused by the *B. microti*-like piroplasm both in dogs from Portugal and in dogs suspected of clinical piroplasmosis outside of Spain.

**Conclusions:**

Although the bitch and the male had visited neighboring Galicia (northwestern Spain), where the disease is endemic, incursion of this piroplasm into northern Portugal is evident and infection of the non-traveled pup was due to either vertical transmission or autochthonous tick infection.

## Background

Species of protozoa from the *Babesia *and *Theileria *genera (order Piroplasmida) infect dogs in many parts of the world and cause important diseases known as babesiosis or piroplasmosis [1]. The etiological agents are transmitted by ixodid tick vectors [2], although transmission via blood transfusion [3] and the placenta [4] have been reported for some babesial species and dog to dog transmission of *B. gibsoni *by dog bites is strongly supported by epidemiological evidence [5-8]. Canine babesiosis may range from being sub-clinical to severe and fatal, depending on the virulence of the pathogen species or strain [9] and also on the susceptibility of the individual host as related to its age, immune status and concurrent infection or illness [1,10]. Lethargy, anorexia, pale mucous membranes, hyperthermia, hemoglobinuria, splenomegaly, hemolytic anemia and thrombocytopenia are clinical manifestations frequently described in dogs suffering from piroplasmosis [11,12].

The size of their pear-shaped intraerythrocytic stages (piroplasms) has traditionally been used for the identification of *Babesia *species in dogs: large forms of *Babesia canis *(3-5 μm) and small *Babesia gibsoni *(0.5-2.5 μm). Additional criteria, especially molecular techniques, have further differentiated several "large" or "small" agents of canine piroplasmosis, including three subspecies of *B. canis *[13] currently regarded as separate species [1,14,15] and one yet unnamed large *Babesia *sp. from North Carolina genetically related to *Babesia bigemina *of cattle [16,17]. *Babesia canis *is the main etiological agent in temperate regions of Europe and causes mild to severe disease [18]. *Babesia vogeli*, the least virulent subspecies, is also present in Europe [12] as well as in tropical or subtropical areas of Africa [19], Asia [20], Australia [21], and North and South America [6,22]. *Babesia rossi*, notoriously the most virulent subspecies, has been reported in western, eastern and southern Africa [23]. *Babesia gibsoni *is present in five continents [1,6,11,20,24], including Europe [25-29]. Other genetically distinct small piroplasms capable of causing disease in dogs are *Babesia conradae*, from California [30], and the canine *Babesia microti*-like "Spanish isolate" or *Theileria annae *(phylogenetically close to zoonotic *B. microti *of humans). The latter is endemic in Galicia, northwestern Spain [31], but was also sporadically found in asymptomatic dogs from Croatia [28] and Mississippi [8].

The increased mobility of dogs may promote the circulation and exchange of vector-borne agents, including canine piroplasms, and their spread into geographical areas where they were previously not endemic [32]. Due to differences in the virulence of babesial species infecting dogs, information on the regional occurrence and prevalence of these agents is important for the diagnosis and management of individual clinical cases. Blood smear examination is useful to distinguish large from small intraerythrocytic piroplasms, but molecular diagnostic tools, such as the polymerase chain reaction (PCR) and DNA sequencing, are more sensitive methods that provide an accurate identification at the species, subspecies or genotype levels [20,33].

Canine babesiosis is endemic in northern Portugal. Ninety six per cent of the molecularly characterized cases of disease have been found infected with large *B. canis *and only 4% with *B. vogeli *[34,35]. However, confirmed cases of small piroplasms in dogs have not been reported in this country. The present study reports three cases of babesiosis in dogs from northern Portugal found infected with small piroplasms.

## Methods

### Dogs and samples

During 2009, blood samples were received at Inno laboratories, in the city of Braga, northwestern Portugal, from three German shepherd dogs clinically suspected of having piroplasmosis: a 4-year old bitch and a 1-year old male (October) and a 2-month female (November). Reported clinical signs included lethargy and pale mucous membranes for all the animals; anorexia for the bitch and the pup; and fever (40°C) for the bitch. The three animals were from the same breeder. The pup had been born from the bitch in late September 2009 and never left Portugal. At the time samples were received, the bitch and pup still lived together, in the district of Braga, in an outdoor environment. Four other littermates had already been taken to other locations and were not available for medical examination. The male dog was also living in an outdoor environment in northwestern Portugal. In early August 2009 the bitch had been taken to Germany to mate and in the middle of that month, on its way back to northwestern Portugal, it was housed for 10 days in a kennel in Vigo, Galicia (northwestern Spain). The male dog had also been housed in the same kennel in Vigo, approximately at the same time. The bitch had been found infested with ticks, whose species was not identified, in the second half of August 2009, after returning to Portugal; the pup never had detectable ticks; and no information on the presence of ticks could be retrieved regarding the male dog.

Blood in EDTA was used to prepare thin glass-slide smears that were air-dried, fixed with methanol, stained with Hemacolor^® ^(Merck, Germany) and then examined under light microscopy (magnification of 1000×) for the detection of possible piroplasms. Blood was also spotted onto individual papers (7.5 cm × 2.5 cm; GB 002 Schleicher and Schuell, Dassel, Germany) allowed to air-dry and stored at -20°C until further use.

A complete blood count (CBC) was performed for all the dogs and repeated CBC was carried out for the bitch two days (day 2) and six days (day 6) after the primary assessment (day 0). A reticulocyte count was done for the bitch on day 2 using new methylene blue (Sigma-Aldrich, UK), as described by Tvedten and Weiss [36]. An additional determination of serum biochemical parameters (total protein, albumin, globulins, urea, creatinine, total bilirubin, glucose, alanine transaminase and alkaline phosphatase) was done on day 0 from the bitch's serum (Prestige 24i; Cormay, Tokyo Boeki Medical System, Japan).

### DNA extraction, PCR and sequence analysis

DNA was extracted from filter papers as previously described [35]. Primers Piro-A (5'-AAT ACC CAA TCC TGACAC AGG G-3') and Piro-B (5'-TTA AAT ACG AAT GCC CCC AAC-3') were used to amplify a 408 bp fragment of the 18S rRNA gene of *Babesia *spp. by PCR [37]. Amplification was done under the following conditions: 94°C for 1 min followed by 39 cycles of 94°C for 45 s, 62°C for 45 s, and 72°C for 45 s. DNA sequencing was performed at the Center for Genomics Technologies, Hebrew University of Jerusalem. Obtained DNA sequences were evaluated with the ChromasPro software version 1.33 and compared for similarity to sequences in GenBank, using the BLAST program hosted by NCBI, National Institutes of Health, USA http://www.ncbi.nlm.nih.gov.

## Results

**Figure 1:**
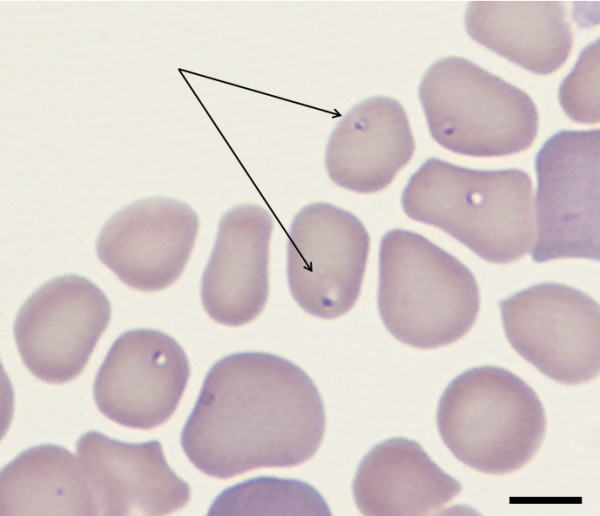
**Intraerythrocytic isolated ring-shaped piroplasms (arrows) of the *Babesia microti*-like small piroplasm in a blood smear from the bitch**. Giemsa; bar = 5 μm.

Intraerythrocytic isolated ring-shaped bodies morphologically compatible with small piroplasms were detected by microscopy in blood smears from the bitch and the pup (Figure 1) but not from the male. Table 1 summarizes hematological results from the three animals. The bitch primarily had a hypochromic normocytic anemia at day 0 that changed to hypochromic macrocytic on day 2. An increasing level of polychromasia was evident in the blood smear at days 0 (1+), 2 (3+) and 6 (4+), as well as other hematological findings compatible with regenerative anemia, i.e. anisocytosis, metarubricytes and Howell-Jolly bodies. A count of 1 × 10^5 ^reticulocytes/μl on day 2 (regarded as a mild to moderate increase, which is compatible with 6 × 10^4 ^to 2 × 10^5 ^reticulocytes/μl), further confirmed regenerative anemia. Toxic neutrophils (10-30%), at days 0 and 2, exhibiting Döhle bodies and cytoplasmic basophilia and vacuolization, and reactive lymphocytes were also observed. Thrombocytopenia was also present and changed from severe at day 0 to mild at day 6. Macroplatelets could be detected, at days 0 and 2, suggesting increased bone marrow megakaryocytic activity. On the biochemical profile, bilirubin concentration was mildly elevated at 0.63 mg/dl (normal range: 0-0.3). Results for the other measured parameters were within normal limits.

**Table 1:** Results from hematological analyses of the three dogs with babesiosis due to the *Babesia microti*-like piroplasm.

**Parameter**	**Bitch**			**Male**	**Pup**	**Normal range**
				
	**Day 0**	**Day 2**	**Day 6**	**Day 0**	**Day 0**	
				
RBC (10^6^/μl)	2.4	1.9	2.3	2.8	1.3	5.5-8.5
Hb (g/dl)	5.4	4.6	5.7	7.4	2.5	12-18
Hct (%)	17.9	17.0	20.6	26.7	8.2	37-55
MCV (fl)	73.7	88.1	88.4	95.9	66.0	60-74
MCHC (g/dl)	30.2	27.1	27.7	27.8	30.5	31-36
RDW (%)	16.5	16.5	16.4	20.6	21.0	12-18
Nucleated RBC (%)	ND	15.0	22.0	81.0	ND	< 3
WBC (10^3^/μl)	10.8	14.0*	7.8*	23.1*	2.8	6-17
Segmented neutrophils (10^3^/μl)	7.8	10.4	5.4	13.6	0.8	3-11.8
Band neutrophils (10^3^/μl)	0.0	0.0	0.0	0.0	0.6	0-0.5
Lymphocytes (10^3^/μl)	2.1	1.8	1.9	8.3	1.2	1-4.8
Monocytes (10^3^/μl)	1.0	1.7	1.1	1.4	0.2	0.2-2
Eosinophils (10^3^/μl)	0.0	0.0	0.2	0.0	0.0	0.1-1.3
Platelets (10^3^/μl)	19	63	165	ND**	54	200-500

Hb: hemoglobin; Hct: hematocrit; MCHC: mean corpuscular hemoglobin concentration; MCV: mean corpuscular volume; ND: not determined; RBC: red blood cells; RDW: red blood cell distribution width; WBC: white blood cells; * WBC count corrected for the presence of nucleated RBC; ** platelet aggregates hindered platelet count.

The male dog presented a moderate hypochromic macrocytic anemia, with findings typical of red blood cells (RBC) regeneration, i.e. polychromasia, 81% of nucleated RBC (metarubricytes and rubricytes) and anisocytosis. Mild leukocytosis (23.1 × 10^3 ^cells/μl) was noted, with mild neutrophilia and severe lymphocytosis. Platelet count could not be done, due to the existence of platelet aggregates, which were detected by blood smear observation.

The pup had severe hypochromic normocytic anemia with polychromasia and anisocytosis. Moderate to severe leukopenia with a degenerative left shift and more than 30% of toxic neutrophils exhibiting Döhle bodies, cytoplasmic basophilia and vacuolization, and reactive lymphocytes were found. Severe thrombocytopenia as well as macroplatelets were observed.

The three animals were all found positive for *Babesia *spp. by the PCR assay. Further sequence analysis revealed that the bitch and male yielded an identical 414 bp long sequence that was 409/414 (98%) identical to the GenBank closest sequence EU583387.1, the canine small *Babesia *"Spanish isolate" or *B. microti*-like piroplasm. The pup yielded a 413 bp sequence that was 409/413 (99%) identical to GenBank EU583387.1 and 99% identical to the bitch and male sequence.

Imidocarb dipropionate (6 mg/kg, subcutaneous injection, repeated 14 days later) was used to treat the bitch (on day 1), the male and the pup. Available information suggested an apparent clinical recovery of the three dogs; however, the pup died in December 2009 due to parvoviral enteritis.

## Discussion

To the best of our knowledge, this is the first report of molecular identification of the *B. microti*-like piroplasm both in dogs from Portugal and in dogs suspected of clinical piroplasmosis outside of Spain.

The designation *B. microti*-like piroplasm has been used in the present description, as genetic analyses revealed that *T. annae *[38] is more closely related to *B. microti*, a rodent piroplasm that causes babesiosis in humans, than to other *Theileria *spp. [8,39,40]. Furthermore, no evidence has been presented for pre-erythrocytic stages of this infection in leukocytes, which is a characteristic of the theilerial life cycle [41]. The name "Spanish isolate" also used to describe this pathogen [8] comes from the first description of this causative agent, which was made from a dog with piroplasmosis (originated from a *Babesia*-free area in Germany) that had travelled to northeastern Spain [38].

The *B. microti*-like piroplasm can cause severe disease and mortality in dogs and is endemic in Galicia, northwestern Spain [31,39], which northerly borders the area where the three dogs from the present report lived in Portugal. According to the published information, the *B. microti*-like piroplasm had been molecularly sequenced from dogs with clinical babesiosis only from Spain and mostly from Galicia [31,39,42]. Besides, there are reports of infection in a few clinically healthy dogs: one from Tarragona, northeastern Spain [43], another one from Croatia [28] and an additional one from Mississipi [8]. DNA of the *B. microti*-like piroplasm has also been detected in foxes from central and northern Spain [44-46], eastern Canada, North Carolina in the USA [47], Croatia [48]; in cats from Portugal [49] and Italy [50]; in a donkey from northern Spain [51]; in roe deer from Italy [52]; in feral raccoons from Japan [53]; in *Ixodes ricinus *and *Rhipicephalus sanguineus *ticks from Italy [54,55]; and in *I. ricinus *and *Ixodes hexagonus *from northern Spain [46]. To date, this piroplasm species has not been reported in Africa or Australia.

*Ixodes hexagonus *is the main candidate vector of the *B. microti*-like piroplasm in Galicia [56]. This tick species has also been found in northern Portugal [57,58], but the endemic nature of the small piroplasm in this area cannot be ascertained. In the present report, the bitch and the male dog were most probably infected during the 10-day period they spend in Vigo (Galicia), although the possibility that the infection originated in Portugal should not be excluded. Both dogs became clinically suspected two months after returning from Vigo. This fact suggests a longer incubation period for the disease caused by the *B. microti*-like piroplasm in comparison with the 4 to 21 days described for other canine babesial infections [2]. However, the pup never left Portugal and could have been infected by vertical transmission. The transplacental transmission of *B. gibsoni *has been experimentally demonstrated in a bitch that delivered a litter of one stillborn and four live pups [4]. These four pups died from congenital babesiosis between 14 and 39 days post-birth.

In horses, once an animal is infected with *T. equi *it remains a lifelong carrier, since anti-theilerial drugs do not completely eliminate the parasite [59]. Infected mares can transmit *T. equi *piroplasms across the placenta and this might result in abortion or neonatal piroplasmosis. Colostral antibodies to *T. equi *may suppress parasitaemia in newborn foals thereby reducing the incidence of clinical neonatal equine piroplasmosis, which could control parasitaemia during the foals' early months of life [59]. In the present report, a more than 2-month time interval between possible infection *in utero *and clinical disease might be explained by a similar protection conferred on the pup by maternal colostrum. Nevertheless, and although it had no detectable ticks, the possibility that the pup was infected after birth by a tick vector cannot be ruled out.

A severe regenerative haemolytic anemia and moderate to severe thrombocytopenia are common findings among dogs infected with the *B. microti*-like piroplasm [60,61]. In the present report, the three dogs had regenerative anaemia, based on reticulocyte count (performed only for the bitch), presence of polychromasia, anisocytosis and nucleated RBC. The mechanisms related with severe hemolytic anemia may be more dependent on the host immune response than on the direct destruction of RBC by the piroplasm [2]. The bitch and pup also had confirmed severe thrombocytopenia, while platelet count could not be assessed in the male dog. Mechanisms of local or systemic intravascular coagulopathy, immune-mediated destruction or splenic sequestration may be implicated in severe thrombocytopenias. On the other hand, the almost constant presence of macroplatelets in blood smears is associated with a bone marrow regenerative response to platelet consumption, as well as sequestration or destruction [2].

The bitch had normal leukocyte counts, while the male dog presented leukocytosis and the pup had leukopenia. Camacho-García [31] describes that half the dogs with piroplasmosis due to the *B. microti*-like piroplasm had normal leukocyte counts. Nevertheless, the values may range between leukopenia and leukocytosis, with the latter reaching extreme counts compatible with a leukemoid response in immune-mediated hemolytic anemia [60]. Many cases also develop serum biochemistry abnormalities compatible with a glomerular component of renal failure [31]. In a study describing 58 infected dogs, 36% were azotemic at the time of diagnosis and 22% died with azotemia being the main cause of mortality [62]. In the present report, serum creatinine and urea were assessed only for the bitch and found to be within their normal range.

According to Camacho-García [31], animals infected with the *B. microti*-like piroplasm had a syndrome clinically more severe than those infected with *B. canis*, treatment with imidocarb dipropionate was less effective and evolution towards renal failure more frequent. At the time the pup died from parvoviral enteritis, one month after diagnosis of babesial infection, it had apparently recovered from the clinical disease caused by the small piroplasm.

The definitive diagnosis of babesiosis for the male dog was achieved only after the PCR and sequencing results. Indeed, in dogs clinically suspected of babesiosis, microscopy may lack sensitivity due to low parasitaemia [1]. Sensitive molecular detection and species identification are important for the selection of the appropriate therapy and for prognosis, as well as for the screening of subclinical infections and blood donors [29].

## Conclusions

This study reports the first confirmed cases of canine babesiosis caused by the *B. microti*-like piroplasm in Portugal involving two adult dogs and a pup. Although the two adult dogs may have been infected in the neighboring Spanish province of Galicia, incursion of this infection into northern Portugal where possible suitable tick vectors are present is evident. Infection of the non-traveled pup was due to either vertical transmission or autochthonous tick infection. Awareness of the risk of spread of the *B. microti*-like piroplasm to additional countries via dog travel or infected vector transport should be increased and efforts to prevent further spread of this infection are warranted.

## Competing interests

The authors declare that they have no competing interests.

## Authors' contributions

Conceived and designed the study: LC and GB. Collected and characterized clinical samples: PBS and MA. Performed PCR and genetic analysis: YYM and GB. Analyzed data, drafted and revised the manuscript: PBS, LC and GB. All authors gave final approval of the version to be submitted.
